# The Role of High Frequency Dynamic Threshold (HiDT) Serum Carcinoembryonic Antigen (CEA) Measurements in Colorectal Cancer Surveillance: A (Revisited) Hypothesis Paper

**DOI:** 10.3390/cancers3022302

**Published:** 2011-05-11

**Authors:** Irene Grossmann, Charlotte Verberne, Geertruida de Bock, Klaas Havenga, Ido Kema, Joost Klaase, Andrew Renehan, Theo Wiggers

**Affiliations:** 1 Department of Surgery, Catharina Hospital Eindhoven, Michelangelolaan 2, 5623 EJ Eindhoven, the Netherlands; 2 Department of Surgery, University Medical Center Groningen, Hanzeplein 1, 9713 GZ Groningen, the Netherlands; E-Mails: c.j.verberne@chir.umcg.nl (C.V.); t.wiggers@chir.umcg.nl (T.W.); 3 Department Epidemiology and Biostatistics, University Medical Center Groningen, Hanzeplein 1, 9713 GZ Groningen, the Netherlands; E-Mails: g.h.de.bock@epi.umcg.nl (G.D.B.); k.havenga@chir.umcg.nl (K.H.); 4 Department of Clinical Chemistry, University Medical Center Groningen, Hanzeplein 1, 9713 GZ Groningen, the Netherlands; E-Mail: i.p.kema@lc.umcg.nl (I.K.); 5 Department of Surgery, Medical Spectrum Twente, Haaksbergertsraat 55, 7513 ER Enschede, the Netherlands; E-Mail: j.klaase@mst.nl (J.K.); 6 The Christie NHS Foundation Trust, School of Cancer, Enabling Sciences and Technology, University of Manchester, Wilmslow Road, Manchester M20 4BX, UK; E-Mail: andrew.renehan@btinternet.com (A.R.)

**Keywords:** carcinoembryonic antigen, colorectal cancer, follow-up, recurrent disease, metastases

## Abstract

Following curative treatment for colorectal cancer (CRC), 30% to 50% of patients will develop recurrent disease. For CRC there are several lines of evidence supporting the hypothesis that early detection of metachronous disease offers a second opportunity for cure. This paper revisits the potential role of serum carcinoembryonic antigen (CEA) in follow-up. A comprehensive review of the literature (1978–2008) demonstrates that the initial promise of serum CEA as an effective surveillance tool has been tarnished through perpetuation of poorly designed studies. Specific limitations included: testing CEA as only an ‘add-on’ diagnostic tool; lack of standardization of threshold values; use of static thresholds; too low measurement frequency. Major changes in localizing imaging techniques and treatment of metastatic CRC further cause a decrease of clinical applicability of past trial outcomes. In 1982, Staab hypothesized that the optimal benefit of serum CEA as a surveillance tool is through high-frequency triage using a dynamic threshold (HiDT). Evidence supporting this hypothesis was found in the biochemical characteristics of serum CEA and retrospective studies showing the superior predictive value of a dynamic threshold. A multi-centred randomized phase III study optimizing the usage of HiDT against resectability of recurrent disease is commencing recruitment in the Netherlands.

## 1. Introduction

Following curative treatment for primary colorectal cancer (CRC), 30% to 50% of patients will develop recurrent disease, commonly locoregional recurrences and distant metastases to the liver and lung. Unlike most adult solid malignancies, for CRC there are several lines of evidence supporting the hypothesis that early detection of metachronous disease offers a second opportunity for cure [1-4].

Serum CEA has been the hallmark in follow-up of colorectal carcinoma for 30 years. There has been considerable controversy concerning the benefits of serum CEA in follow-up. Five meta-analyses were carried out analyzing the evidence of the effectiveness of follow-up in colorectal cancer on survival [1-5]. Only a small survival gain was seen, predominantly when frequent serum CEA measurements were included in the follow-up [1-4]. Most of the studies included in the meta-analyses however analyzed the effect of follow-up in general, often consisting of a wide variety and combinations of diagnostic tests [3]. This limits the evidence on the diagnostic accuracy of a specific diagnostic test. The meta-analyses therefore do not necessarily reflect the diagnostic value of serum CEA in follow-up and the controversy concerning the role of serum CEA in follow-up has not been conclusively solved.

The current drive in clinical trials is towards detecting metachronous disease in asymptomatic patients through more intensive imaging protocols, typically computerized tomography (CT) scanning, but this has several limitations including: highly expensive and resource intensive; high-rates of ‘incidentaloma’ incurring further costs for investigations and treatments; and neoplastic risks from cumulative radiation exposure.

Against this background, an alternative strategy is a low cost ‘triage’ blood biomarker triggering and directing selective CT usage. This paper revisits the potential role of serum carcinoembryonic antigen (CEA), set against modern imaging and CRC treatment and clinically-linked decision-making software, as such a surveillance tool.

## 2. Limitations of Past Studies

A comprehensive review of the literature (1978–2008) was performed, using search term [Carcinoembryonic antigen] AND [Colorectal neoplasm], limited to the subheadings [analysis], [diagnostic use], [blood], [standards] and limited to ‘human’ and ‘English language’. Additional relevant references found in articles were included. Review articles were used as a reference but not included in the analysis. Selection of the abstracts was made on the available information concerning clinical use of CEA in follow-up after curative resection of CRC.

From 1980 until 2008, 26 original clinical trials evaluating the outcome of follow-up after curative treatment of colorectal carcinoma which included serum CEA measurements were published (Table 1) [6-31]. One publication was excluded from analysis because it concerned a double publication. These 25 clinical trials, and a few follow-up studies that did not include serum CEA measurements, have resulted in five meta-analyses on follow-up in CRC [1-5]. The 25 original articles were analyzed on actual testing of the diagnostic capacity of serum-CEA, the clinically applied threshold value of serum-CEA, timing of follow-up and measurement frequency.

Most studies evaluated follow-up regimes consisting of various diagnostic tests. In only five of 25 clinical trials, *different* serum CEA regimes were compared [6-10], from which three studies were randomized controlled trials [8-10]. The results of these studies show a consistent beneficial effect of serum CEA measurements on both the rate of curative resection of recurrent disease and survival (Table 2). In the meta-analyses the majority of included trials did not compare *different* CEA regimes (Table 3). These meta-analyses therefore, do not reflect the specific diagnostic capacity of serum CEA in follow-up.

The static normal value of serum-CEA as advised by manufacturers is 2.0–2.5 ng/mL, depending on the actual test. To increase specificity and prevent the situation of inability to localize recurrent disease with imaging techniques or relaparotomy in the past, the *clinically* applied static threshold value is often increased to 5 ng/mL or higher. From the 25 studies, all except one study used a static threshold value. A threshold value of ≤2.5 ng/mL was applied in three studies (13%), of ≥5 ng/mL in eight studies (32%) and in 14 studies the applied threshold value was not mentioned. Applying a higher threshold value, results in a decrease in sensitivity together with a possible delay in diagnosis of recurrent disease.

Diagnostic tests for recurrent disease are most sensitive when the timing is aimed at the expected time to recurrence through optimizing the pre-test probability. Data from the 25 clinical studies revealed that local recurrences were found on average between six months and two years, liver metastases between six and 18 months and pulmonary metastases between 24 and 36 months. In the clinical trials (calculation based op 22 trials), a measurement frequency of ≤3 months was attained in 91% of trials in the first year, 53% in the second year and 25% in the third year. The measurement frequency *actually* carried out is only 46% to 62% of the *reported* measurement frequency, as was shown in six independent studies [7,13,22,32-34]. This implies that the timing and measurement frequency in most studies and in clinical practice has been insufficient to expect any beneficial effect of serum-CEA in follow-up for CRC.

## 3. Historical Context: Changes in Clinical Practice

### 3.1. The Role of Imaging Techniques

Localization of recurrent disease in follow-up has been a major problem in the past, influencing the results of clinical trials carried out before 2000. Serum CEA can signal recurrent disease as a first-line screening tool, but for the definite diagnosis and treatment, the site and extent of recurrent disease must be localized. When recurrent disease is suspected but no certainty or treatment can be offered to the patient, follow-up only results in a loss of life quality and should be prevented. Up until this century, is has been very difficult to localize recurrent disease with non-invasive techniques. A second look laparotomy aimed at potentially curable intra-abdominal metastases has been standard of care until the late eighties and has been the hallmark in most clinical trials included in this analysis. Because of the invasive nature of this ‘diagnostic test’, the demands on accuracy of the screening test have been high. Nowadays, non-invasive tests such as multislice computed tomography (CT), contrast-enhanced ultrasound and Positron Emission Tomography (PET) can localize recurrent disease of >1 cm in liver and lung and locoregional recurrence. This situation lowers barriers to act upon signals indicating possible recurrent disease from first-line screening with serum-CEA. The discriminative capacity of available imaging techniques however is limited for smaller lesions. When serum CEA is used in follow-up as a first line detector of recurrent disease, the applied threshold value should be adapted to the likelihood of actual localization on imaging techniques. A threshold of at least 50% detected lesions on imaging following disconcerting serum CEA test results should be attained. This is to prevent recurrence of the ‘old’ problem of inability to localize recurrent disease which results in the inability to offer treatment or support.

The reverse situation that may occur is recurrent disease that does not result in an increase in serum CEA value. A significant proportion of patients have normal serum CEA values with CRC or recurrent disease. This was reviewed in three studies [23,35,36]. The proportion varied from 23% to 60%, depending on the clinically applied (static) threshold value. When respecting a threshold value of 2.5 ng/mL, the proportion of patients with normal CEA values is approximately 25% at initial presentation. Because of different tumor behavior, the proportion of serum CEA ‘negative’ disease is probably lower in recurrent disease; however this has not been described. It is widely believed that a normal serum CEA value, at initial presentation, implies that the serum CEA value will also not rise in case of recurrent disease. Although there is indeed a tendency for serum-CEA ‘negative’ tumors to less often show an increase in serum CEA value with recurrent disease, more than 60% will show a rise with recurrent disease [36]. Serum CEA measurements can therefore not be discarded based on an initial normal CEA value. However, there will always remain patients that have recurrent disease with normal static serum CEA values. Any CEA based follow-up protocol should therefore include imaging techniques, aimed at the expected time of recurrent disease.

### 3.2. Logistic Considerations

One of the reasons that may have contributed to the striking absence of clinical trials applying more frequent serum CEA measurements (at least every 3 months or as recommended by Staab every 6–8 weeks) may be due to the logistic burden and cost of intensive screening. Colorectal cancer is the second most common malignancy with a life time risk of more than 10% in the Western population [37]. Outpatient clinic visits as frequent as the suggested CEA measurements for all patients treated with curative intent is hardly feasible, nor effective; outpatient clinic visits with physical examination do not contribute to early detection of recurrent disease [22] and it can be an unnecessary burden for the (elderly) patient. This situation may have contributed to the notable low adherence to current guideline recommendations on the measurement frequency of serum CEA as well. The solution to these logistic problems is serum CEA measurement *without* outpatient clinic visits. This can nowadays be supported with automated processing, signaling and communication of test results with the patient via postal mail, telephone or internet.

### 3.3. Discussion

Summarizing, the diagnostic accuracy of serum CEA has been underestimated because of the methodology of clinical trials and meta-analyses. Evidence supporting the clinical value of serum CEA on increasing the eligibility of curative resection of recurrent disease detected in asymptomatic patients and successively on survival is available. The threshold value, measurement frequency and interpretation of serum CEA values, and the expected time of recurrent disease have been far from optimal in the past in both clinical trials and clinical practice. The changing clinical context limits the value of the outcomes of past clinical trials. Curative treatment options for recurrent disease have been improved considerably in the last decade, and more accurate non-invasive imaging techniques and computer support in medical care are available, supporting intensive follow-up based upon serum CEA measurements in colorectal cancer.

## 4. Revisited Hypothesis

Serum CEA reflects the expression of an embryonic protein on the cell surface as an antigen response, which has a function in the embryonic tissue development. This protein is expressed in the adult situation in malignancies and inflammatory conditions. There is a relation between tumor growth and serum CEA; most tumors have an exponential growth pattern followed by an exponential rise in CEA [35,38]. Following these observations, Staab hypothesized in 1982 that the optimal benefit of serum CEA as a surveillance tool is through high-frequency triage using a dynamic threshold [15,39].

This hypothesis was revisited by means of a comprehensive review of the literature and acting upon changes in the clinical context of colorectal cancer. The revised concept (abbr. HiDT) comprised of monthly serum CEA measurements applying a dynamic threshold value (proportional rise), combined with yearly CT scanning and yearly outpatient clinic visits. Successively this concept was tested in a phase II trial, and the initial results are herein discussed.

### 4.1. Biochemistry, Sensitivity and Specificity of Serum CEA

Serum CEA measurement tests are based on monoclonal antibodies that bind CEA and, as with any diagnostic test, has measurement errors. Standard measurement errors of serum tests are divided into four types. The intra-assay test variation, that is the variation in test result when using the same blood sample, is approximately 2%–10%. The inter-assay variation, that is the variation in test result using the same blood sample in two different tests, is 4–12% in the lower ranges (<15 ng/mL) and up to 20% in higher ranges [40]. The intra-individual variation, that is the variation in serum CEA values within one person without evidence of disease, is 9.3%. The inter-individual variation, that is the difference in normal value between individuals, is approximately 55% [41]. Referencing the CEA value on the patient's previous value can eliminate this last and largest measurement error. This requires repeated measurements and interpretation of the changes in serum CEA value.

Because of the other three measurement variations, the threshold value for rise should not be lower than 15%. The static threshold value as recommended by industrial standards ranges from 2.0–2.5; the optimal dynamic threshold has not yet been formulated.

The *clinically* optimal threshold value is dependent on the desired sensitivity and specificity in the target population. Sensitivity must be high in follow-up to adequately detect recurrent disease. A high specificity is of importance to minimize unnecessary diagnostic evaluation based on test results, especially when the test is applied as a screening tool. Follow-up requires both, as a screening tool in a high-risk population. The specificity increases when the pre-test probability is high; meaning the test is most accurate in a high-risk population. In four studies the sensitivity and specificity of serum CEA on static threshold values ranging from 2.5–20 ng/mL was calculated [13,20,26,42]. The pooled results show a consistent pattern of a low accuracy between 2.5 and 10 ng/mL. This is predominantly caused by the overlap of abnormal CEA values with benign disease in these lower ranges and the inter-individual variation. The difference between malignancies and inflammatory conditions is that malignancies will continue to grow where inflammation is usually self-limiting. The accuracy can be improved by using intra-individual rise, because this eliminates the inter-individual variation and shows the potentially discriminative *pattern* of rise. This requires repeated measurements and consequently a high measurement frequency. The optimal measurement frequency for this approach has been addressed in three studies [6,15,43]. Based on their retrospective data, all suggested a frequency in the order of every one to two months. In summary, on evaluating the rise-pattern, the inter-individual variation can be eliminated and a higher discriminative capacity between malignancy and inflammatory conditions is expected. This increases specificity and allows interpretation of serum CEA values in lower ranges of values, thus increasing sensitivity as well. In short; interpretation of serum CEA values with a dynamic threshold based upon high frequency measurements (HiDT) can largely improve accuracy.

### 4.2. Evidence of the Hypothesis in Past Clinical Trials

A search with [Carcinoembryonic antigen] limited to subdivisions *analysis, *diagnostic use, *blood and *standards AND ‘doubling time’, and a search with [Carcinoembryonic antigen] AND [Colorectal neoplasm] AND ‘doubling time’ or ‘rise’ was done, both limited to ‘human’ and ‘English language’. Relevant references were included in the study. Abstracts were selected on information on clinical relevant information on use of a dynamic threshold value of CEA. Twelve articles were included [13,15,35,38,39,43-49] and analyzed on study design, clinical effectiveness and quantitative results on CEA rise found with recurrent disease.

The hypothesis on using a dynamic threshold value was evaluated in a few studies. In the study of Steele, the predictive capacity of using a static *versus* dynamic threshold value (of >3% rise per month) was compared in a retrospective analysis of 767 patients with CRC [13]. The proportion of patients with recurrent disease *without* rise varied from 10%–27%, the proportion of patients with recurrent disease *with* rise varied from 33%–84% (variation dependent on the lower limit static threshold value). The dynamic threshold value (rise) had a much stronger predictive capacity for recurrent disease than the actual height of the serum CEA value, supporting the hypothesis on HiDT. Another trial recalculated in retrospect the pattern of rise and found a median increase of 20% per month in case of recurrent disease as compared to median rise of 0.3% in recurrence free patients [44]. Only one clinical trial applying CEA-rise in follow-up has been carried out by Staab [39]. This study showed a modest survival benefit after three years (5% *versus* 25%), comparing patients that had, or refused, relaparotomy induced by CEA rise. This study does not *prove* a better outcome when applying a dynamic threshold value in CEA as compared to other follow-up methods; however it does suggest that early detection and treatment of recurrent disease may result in a survival benefit. Further, it was carried out in a time when non-invasive imaging techniques to localize recurrent disease were not yet available. Most studies on CEA rise or doubling time, evaluated the extent of rise with recurrent disease that may help the determination of the accurate threshold value [13,35,43,44,46-49] (Table 4). None analyzed the pattern of rise and decrease with inflammatory conditions or in smokers.

### 4.3. Results from the Phase II Trial

A phase II trial was started in 2008 evaluating the clinical applicability and outcomes of HiDT serum CEA measurements in follow-up after curative treatment of colorectal cancer. The primary endpoint is the eligibility of curative treatment of recurrent disease (increase to 30%), secondary endpoints are the ability to localize recurrent disease on imaging techniques (CT, minimum 50%) and feasibility of the computer supported protocol. This study was done in two different hospitals. One hospital (n = 64) applied monthly serum-CEA measurements with a dynamic threshold value of >10% rise each month in two consecutive measurements. The other hospital (n = 177) applied serum CEA measurements every three months, that was repeated after six weeks in case of a rise of >10%. In both hospitals a routine CT of the abdomen was performed at year 1 and a CT of chest and abdomen at year 2. Patients with American Joint Committee on Cancer (AJCC) stage II and III colorectal cancer treated with curative intent were eligible for inclusion. The median follow-up time at interim analysis was 18 months.

**Figure 1.**
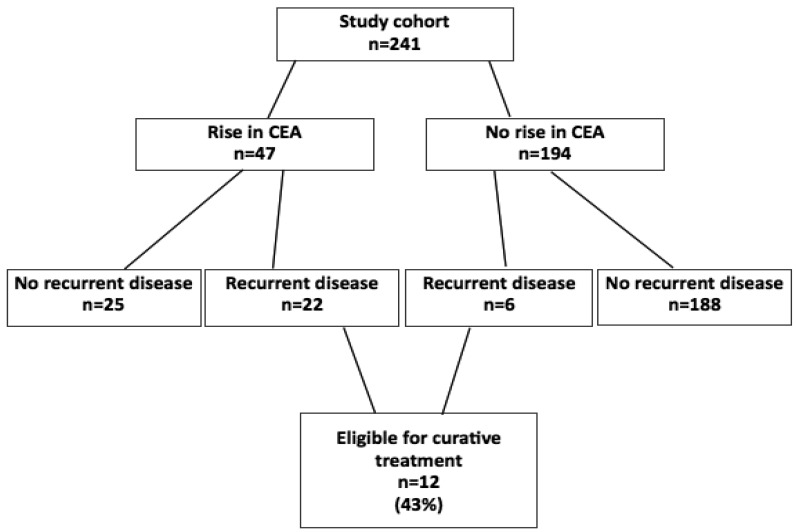
Results from the phase II trial.

Recurrences after curative treatment, until now, were found in 28 patients (12%). Of 28 patients with recurrent disease, 12 patients (43%) were eligible for curative treatment (Figure 1). The sensitivity of HiDT serum CEA measurements was 79% and the specificity was 88% in a range between 2.5 and 10 ng/mL. Mean increase factor per month was 1.48 in the recurrence group. In a subgroup analysis of patients from the first hospital who had false positive rises in CEA values (n = 8), other causes, such as inflammatory disease (n = 3) and dysplastic polyps (n = 2), were found as the cause of the rise; in these patients CEA values decreased after treatment. The mean increase factor per month in the group of patients with false positive rises in CEA was 1.25. The dynamic threshold value applied in the study is probably too low; approximately half of the patients with a rise in CEA did not have recurrent disease. A solution for this problem—Besides alteration of the threshold value—is to focus additional diagnostics on possible recurrence, inflammatory diseases and dysplastic polyps.

A subgroup analysis of the effect of smoking on base CEA values was done in one hospital (n = 44). The mean base CEA value for smokers who did not show a rise in CEA (n = 11) was 5.1, while the mean base value of non-smokers without rise in CEA (n = 23) was 2.9 ng/mL. The non-smokers with recurrent disease (n = 5) showed a mean rising factor of 1.32 per month, and the smokers (n = 2) showed a mean rising factor of 1.39 per month.

These preliminary results show an improved accuracy when applying HiDT approach and had an acceptable outcome concerning the primary and secondary endpoints.

Prototype software (CEA Watch) has been designed and the initial experience was good [50]. Patients' experiences with HiDT with reduced outpatient clinic visits was evaluated in a psychological side-study, showing this approach is well tolerated and even preferred by patients over care as usual [51].

## 5. Phase III Trial Design

A multi-centered randomized phase III study on the usage of HiDT of serum CEA as a low cost ‘triage’ blood biomarker triggering and directing selective CT usage, compared to care as usual is commencing recruitment in the Netherlands in 2011. All patients after curative treatment of AJCC stage II-IV colorectal cancer and fit to undergo major surgery for recurrent disease are eligible for this study. The intervention will be bi-monthly serum CEA measurements applying a dynamic threshold value of 20% rise and yearly CT of chest and abdomen at year 1, 2 and 3. The control group is care as usual. A clinically integrated computer-assisted support system is a key component of the trial design and will concurrently be further evaluated. The study will be performed in 12 hospitals using a stepped wedge design. Different clusters crossover at different time points in one direction—From control to intervention. The *time* at which the cluster may start the intervention is randomized, and the baseline starting point is January 1st 2011. The primary outcome measure is the eligibility of intended curative treatment of recurrent disease. Secondary outcome measures are: overall and disease free survival; optimizing the threshold value set against attainability of localizing recurrent disease on imaging; psychological effects and logistic effectuation of HiDT serum CEA measurements.

## 6. Conclusions

With modern multi-modality treatment options, the chances on curation of metastatic or recurrent CRC are continously improving, and motivate a new effort to optimize oncological follow-up. The potential role of serum CEA as a first-line surveillance tool directing selective CT usage, was evaluated in this review. It shows that the benefits of serum CEA in follow-up have been underestimated by perpetuation of past poor trial designs and insufficient clinical use. Modern imaging techniques provide the opportunity to localize suspected recurrences at an early stage. High frequency measurements and a dynamic threshold value (HiDT) increases both the sensitivity and specificity of serum CEA. The outcomes of a phase II trial applying the HiDT approach were encouraging, in an increase of eligibility for curative treatment towards 40%, good experiences with the clinically-linked decision making software and a higher patient satisfaction. These outcomes have set forth a national randomized clinical trial that is currently commencing recruitment in the Netherlands.

## Figures and Tables

**Table 1.** Clinical studies on follow-up including CEA measurements (n = 25).

	**Year**	Type trial**	**N=**	**Recurrence rate**	

				**N=**	**%**
Martin	1980	Prospective, Comparative	300	60	20%
Lim	1980	Retrospective, Non-comparative	127	20	16%
Steele	1982	Retrospective, Non-comparative	770	86	11%
Minton	1984	Prospective, Comparative	400	130	33%
Hine	1984	Prospective, Non-comparative	626	171	27%
Staab	1985	Prospective, Non-comparative	426	67	16%
Ovaska*	1990	Retrospective, Non-comparative	507	149	29%
Behbehani	1990	Prospective, Non-comparative	123	34	27%
Moertel	1993	Retrospective, Non-comparative	1216	417	34%
McCall	1993	Prospective, Non-comparative	311	98	32%
Makela	1995	Prospective, Non-comparative	106	43	41%
Ohlsson	1995	RCT, Comparative	107	35	33%
Pietra	1998	RCT, Comparative	207	46	22%
Schoemaker	1998	Prospective, Non-comparative	325	?	?
Graham	1998	Retrospective, Non-comparative	1356	421	31%
Wichmann	2000	Prospective, Non-comparative	1321	306	23%
Komborozos	2001	Retrospective, Non-comparative	113	113	100%
Bleeker	2001	Prospective, Non-comparative	496	213	43%
Glover	2002	Retrospective, Non-comparative	100	32	32%
Secco	2002	RCT, Comparative	337	184	55%
Chau	2004	Prospective, Non-comparative	530	154	29%
Bonthuis	2004	Retrospective, Non-comparative	564	149	26%
Grossmann EM	2004	RCT, Non-comparative	985	139	14%
Rodriquez	2006	RCT, Non-comparative	259	69	27%
Fernandes	2006	Prospective, Non-comparative	120	23	19%

* Results of this study were published twice, therefore only one study is taken into analysis;

** Concerning CEA: all grey-marked studies were comparative concerning CEA.

**Table 2.** Effect of CEA measurements in FU on resectability of recurrent disease and 5 year survival.

	**Difference in CEA measurements (months)**		**Recurrence rate**		**Curative resection recurrent disease**		**5 year survival**	
	**Control**	**Intensive**	**Control**	**Intensive**	**Control**	**Intensive**	**Control**	**Intensive**
Secco	none	3 (HR) 6 (LR)	57%	53%	16%	31%	32% (HR) 60% (LR)	50% (HR) 80% (LR)
Pietra	6	3	19%	25%	10%	65%	58%	73%
Ohlsson	none	3	33%	37%	17%	40%	67%	75%
Martin	3-6	1-2	7%	13%	27%	60%	9%	*
Minton	none	1–2 3–4 >4	20%	28% 29% 61%	12%	54% 23% 13%	10%**	33%

FU: Follow-up.

* at the time of publication the FU time varied from 0–4 years, after which 58% of patients were still alive;

** The 5 year disease free survival between FU with CEA every 1–2 months as compared to “any less frequent interval”.

**Table 3.** Summary of meta-analyses on follow-up for colorectal cancer and inclusion of trials comparing *different* CEA regimes.

	**Year**	Studies*	**+ CEA**	**Conclusions**
Bruinvels	1994	7	3	A significant increase on survival is found only when CEA assays are included in follow-up.
Kievit**	2000	14	3**	Overall survival gain by intensive follow-up varies between 0.5%–2.0%. No sub-analysis on the role of CEA.
Renehan	2002	5	2	Intensive follow was associated with a reduction in all cause mortality (combined risk 0.81, 95% CI 0.70–0.94). The effect was most pronounced when computed tomography and frequent measurements of CEA were used (RR 0.73 95% CI 0.6–0.89)
Jeffery	2002	5	2	There was evidence that an overall survival benefit exists for patients undergoing more intensive follow-up (OR 0.67, 95% CI 0.53–0.84). Because of the wide variation in the follow-up programs used, it wass not possible to ascertain the best combination and frequency of clinic visits and additional investigations from the data.
Tjandra	2007	8	3	Intensive follow-up after curative resection of colorectal cancer improved overall survival and reresection rate for recurrent disease. Regular surveillance with CEA (p = 0.0002) and colonoscopy (p = 0.04) demonstrated a significant impact on overall mortality.

* Total of randomised controlled trials and non-randomised comparative trials;

** From 4 clinical studies it could not be retrieved whether CEA regimes were different and compared, in 7 studies no different CEA regimes were compared.

**Table 4.** Quantitative rise of CEA in patients with recurrent disease.

	**Year**	**Nr pat**	**Nr RD**	Doubling time*	Rise per 30 days*	**Absolute rise**
Steele	1982	767	469		1.5%–15.2%	
Staab	78–85		114	10–231 days	-	
Boey	1984	146	51		20%	
Staab	1985	667	78	-	0.6–4.4 ng/mL	
Carl	1993	259	163	74–164 days	-	
Umehara	1993		31	60 (18–153) days	-	
Korenaga	1997		17	86 (±18) days	-	
Yamamoto	2004		36	41–110 days	-	
Irvine	2007	139	46	-	-	>1 ng/mL above 1st post-operative level
Tanaka**	*2008*		*43*	*150 days*	-	

* when a differentiation per type of metastases was made, the lower and upper limits are given from all analyses on possible curable metastases (liver, lung and peritoneal metastases or local recurrence);

** In this study the threshold value of CEA-DT as a prognostic factor was calculated: no data were available on average CEA-DT in patients with recurrent disease.
